# Resistance to fresh and salt water in intertidal mites (Acari: Oribatida): implications for ecology and hydrochorous dispersal

**DOI:** 10.1007/s10493-013-9681-y

**Published:** 2013-03-01

**Authors:** Tobias Pfingstl

**Affiliations:** 1 Institute of Zoology, Karl-Franzens University Graz, Universitaetsplatz 2, 8010 Graz, Austria; 2 Bermuda Institute of Ocean Sciences Inc. (BIOS), 17 Biological Lane, St. George’s, GE 01 Bermuda

**Keywords:** Inundation, Thalassobiont, Plastron, Biogeography, Bermuda

## Abstract

The resistance to fresh water and seawater in three intertidal oribatid mite species from Bermuda, *Alismobates inexpectatus*, *Fortuynia atlantica* and *Carinozetes bermudensis*, was tested in laboratory experiments. Larvae are more sensitive to fresh and salt water, nymphs and adults showed equal tolerances. *Fortuynia atlantica* and *A. inexpectatus* were more resistant to salt water whereas *C. bermudensis* survived longer in fresh water. Differences in the resistance to fresh and salt water among the three species may be related to their different vertical occurrences in the eulittoral zone but also to the ability of single species to dwell in periodically brackish waters. In all three species half of the specimens survived at least 10 days in fresh water and more than 18 days in salt water. Maximal submersion time in fresh and salt water ranged from 40 to 143 days. Based on median lethal times it could be estimated that each species would be able to survive transport in seawater along the Gulf Stream over a distance of 3,000 km, from Central America to Bermuda. Thus hydrochorous dispersal should be assumed as the most likely mode of dispersal in intertidal fortuyniid and selenoribatid mites.

## Introduction

The colonization of remote islands by flightless arthropods has been a matter of certain scientific investigations (e.g. Schatz 1991; Peck 1994; Coulson et al. 2002; Pugh 1997, 2003) and there are several possible answers to how they reached these isolated environments. Dispersal by wind, named anemochory, bird-mediated transport, also known as zoochory, or transport by water, hydrochory, are the prevalent explanations. Intertidal dwelling arthropods are daily exposed to the wave action of the surf and are thus prone to get washed away into the open ocean. For these organisms, transport via ocean currents may be the most likely mechanism for long-distance dispersal. There are at least two ways to test this conjecture; the first would be to sieve water from the ocean’s surface and examine flotsam for drifting animals, as was done by Peck (1994), and the other would be to test the ability of these organisms to survive long periods submersed or floating in seawater (Coulson et al. 2002). The above mentioned authors, using these methods, confirmed possible hydrochorous dispersal for flightless arthropods, i.e. Acari and Collembola, but further studies are lacking and experimental data are still largely fragmentary. More specific investigations of intertidal target species living on oceanic island are needed to get a clearer picture of long-distance transport mechanisms.

With a distance of 960 km to the closest landmass (Thomas 2004), the archipelago of Bermuda represents such a group of remote islands in the Atlantic Ocean. The geological age of this archipelago is supposed to be approximately 30 million years, but terrestrial life on Bermuda is presumable relatively young as this volcanic landmass definitely rose above sea level 900,000 years ago (Thomas 2004). Recently it was shown that Bermuda harbours a high diversity of intertidal fortuyniid and selenoribatid mites (Pfingstl and Schuster 2012a, b). The biogeographical origin of these animals is unknown and presently only a matter of conjecture, but congeners have been found in Central America and the Caribbean (Schuster 1989, Pfingstl unpubl.). They feed on intertidal algae and dwell exclusively in the eulittoral zone of the intertidal fringe (Krisper and Schuster 2008). Although these mites are primarily terrestrial animals, they have plastron mechanisms (Pugh et al. 1990; Pfingstl and Schuster 2012a, b) and spend nearly half of their lives submerged in seawater and thus are daily exposed to wave and tidal action. In order to assess the possibility of hydrochorous dispersal of these organisms to and from this archipelago, I tested the ability of three local species, *Alismobates inexpectatus* Pfingstl and Schuster, 2012, *Fortuynia atlantica* Krisper and Schuster, 2008 and *Carinozetes bermudensis* Pfingstl and Schuster, 2012, to survive continued submersion in salt water.

Furthermore, Schuster (1979) demonstrated that certain thalassophile oribatid species are still more tolerant to fresh water than to salt water and he argued that this circumstance may be correlated with the ability of these mites to colonize brackish waters, as well. As one of the three species from Bermuda was also found in periodically brackish water bodies, the tolerance to fresh water was also tested in the three species.

## Materials and methods

Samples of intertidal algae were collected during low tide from diverse coastal locations on the archipelago of Bermuda in the years 2011 and 2012. Specimens of the species *A. inexpectatus*, *F. atlantica* and *C. bermudensis* were extracted with a Berlese–Tullgren apparatus. As heat extraction causes stress and may impact on the animals, only the most active individuals were selected for the submersion experiments in order to reduce the risk of using already physically damaged or weakened individuals.

Species were separated into the ontogenetic stages “larva”, “nymph” and “adult” and 20 individuals of each stage were submersed in fresh water and further 20 in salt water. In sum a total of 120 specimens of each species was tested (20 larvae/fresh water, 20 larvae/salt water; 20 nymphs/fresh water, 20 nymphs/salt water; 20 adults/freshwater, 20 adults/salt water). Fresh water and salt water, used in the experiments, were taken from the respective taps provided in the Bermuda Institute of Ocean Sciences, BIOS. The fresh water was not chlorinated and the salt water was pumped from the sea without treatment (salinity of seawater approx. 35 ‰). Individuals were put separately or together into cylindrical plastic vials, filled with fresh/salt water. All experiments were performed in a laboratory at room temperature of approx. 26 ± 1 °C. This temperature coincides with natural conditions, i.e. average water temperature of the Caribbean Sea (supposed place of origin), including the Gulf Stream (supposed vehicle), is in accord with this value and water temperature on Bermuda ranges from 20 to 30 °C during the year. Photoperiod was not omitted.

*Carinozetes bermudensis* retains a conspicuous layer of air to the body surface when flooded (Pfingstl and Schuster 2012b). Accordingly these specimens are positively buoyant and are prone to float on the water surface. Even when they were completely immersed for a certain time and lost grip to the substrate, they floated to the water surface. In order to provide grip for these mites and keep them underwater, the bottom of each vial was lined with filter paper.

The boxes were opened and checked every day under the dissecting microscope. Inactive animals were stimulated with a fine brush and observed over a 5 min interval before they were finally recorded as dead. In most cases dead animals could be easily identified by widely opened anal plates and extruded spermato- or ovipositors and mouthparts.

Median lethal time (LT_50_) was defined as the period of time in which half of the submersed animals have died (e.g. LT_50_ = 20 days → after 20 days 30 of 60 animals have died).

## Results

### *Alismobates inexpectatus*: reaction to submersion

**Fig. 1:**
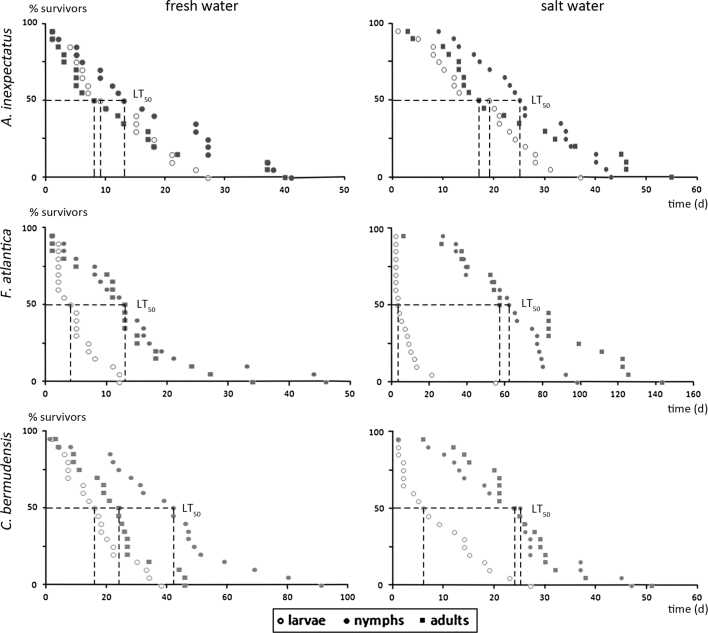
Stage-dependent survival of *Alismobates inexpectatus*, *Fortuynia atlantica* and *Carinozetes bermudensis* in fresh and salt water. *Dashed lines* show interpolated median lethal times (LT_50_)

Nymphs exhibit in fresh and salt water the highest LT_50_ values with 13 days in fresh water and 25 days in salt water (Fig. 1; Table 1). In fresh water larvae and adults showed nearly the same LT_50_ values with 9 and 8 days respectively. In salt water the median lethal time of the larvae is 19 days whereas that of the adults is only 17 days. In fresh water maximum survival time of the adults is nearly identical with that of the nymphs but in salt water one adult survived 12 days longer than the longest persisting nymph.

**Table 1:** Survival times (*Min* minimum, *LT*_*50*_ median lethal time, *Max* maximum) for *Alismobates inexpectatus*, *Fortuynia atlantica* and *Carinozetes bermudensis* in fresh and salt water

	Fresh water			Salt water		
	Min	LT_50_	Max	Min	LT_50_	Max
*A. inexpectatus*						
Larva	1	9	27	1	19	37
Nymph	1	13	41	9	25	43
Adult	1	8	40	3	17	55
Total	**1**	**10**	**41**	**1**	**21**	**55**
*F. atlantica*						
Larva	1	4	12	2	3	55
Nymph	1	13	46	27	62	98
Adult	1	13	34	6	57	143
Total	**1**	**11**	**46**	**2**	**40**	**143**
*C. bermudensis*						
Larva	2	16	38	1	6	27
Nymph	1	42	91	1	25	47
Adult	3	24	46	6	24	51
Total	**1**	**24**	**91**	**1**	**19**	**51**

Stage-independent results are marked bold

The general resistance to salt water, in this species, is slightly greater in each subsequent stage. Independent of stage, the animals die approximately 10 days earlier in fresh water than in salt water.

### Survival of *Fortuynia atlantica*

Larvae show basically the least resistance with a LT_50_ of 4 days in fresh water and 3 days in salt water (Fig. 1; Table 1). In fresh water nymphs and adults exhibit an identical median lethal time of 13 days, in salt water nymphs die later than adults, showing a LT_50_ value of 62 days, whereas half of the adults died in 57 days. Maximum survival in fresh water was shown by a nymph with 46 days and in salt water by an adult with 143 days.

The resistance of larvae to fresh and salt water exhibits no obvious differences but stage independent survival is higher in salt water, this is also shown by the maximum value (stage independent) in fresh water being nearly equal with the LT_50_ value in salt water.

### *Carinozetes bermudensis*: reaction to submersion

Larvae exhibit the least resistance to fresh and salt water with a median lethal time of 16 days in fresh water and 6 days in salt water (Fig. 1; Table 1). Nymphs survived approximately twice as long as larvae and adults in fresh water whereas they showed the same survival as adults in salt water with nearly identical LT_50_ values of 25 and 24 days respectively. Adults showed similar survival in both types of water, but independent of the stage, this species survives longer in fresh water, which is also shown by strongly diverging maximum values.

### Species comparison (Fig. 2)

*Alismobates inexpectatus* and *F. atlantica* are equally tolerant to fresh water, whereas *C. bermudensis* survives longer in this type of water (Table 1). In salt water *A. inexpectatus* and *C. bermudensis* show similar survival times, whereas *F. atlantica* is much more resistant to this type of water than the other two species.

**Fig. 2:**
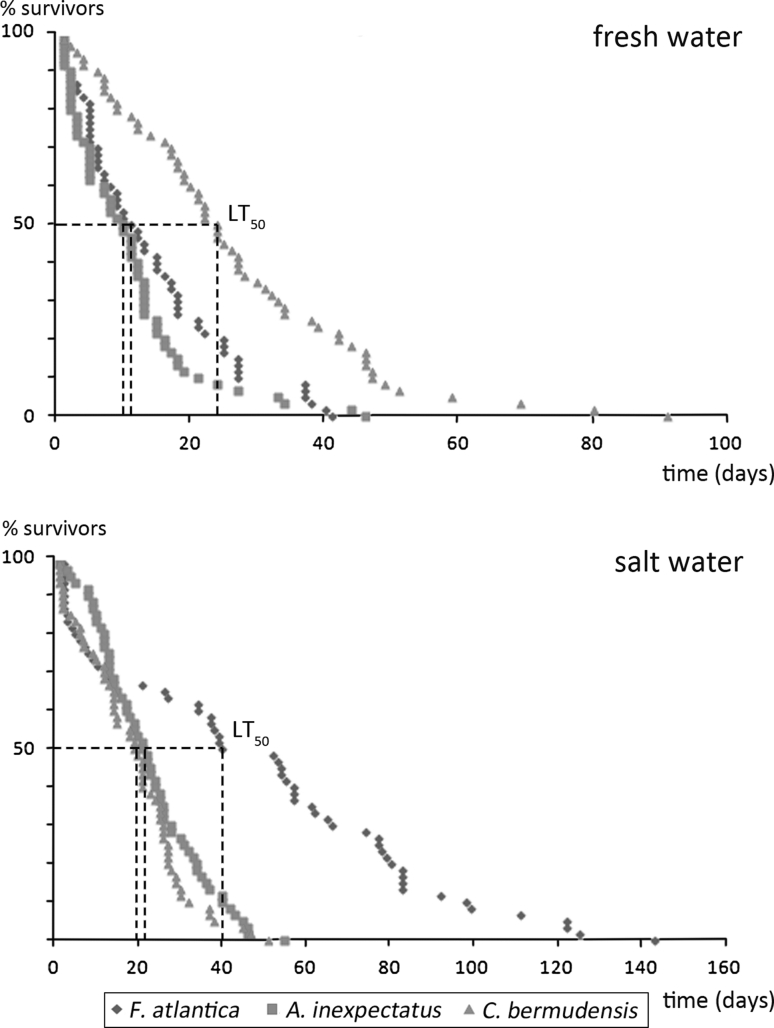
Graphical comparison of the survival of *Alismobates inexpectatus*, *Fortuynia atlantica* and *Carinozetes bermudensis* in fresh and salt water. *Dashed lines* show interpolated median lethal time (LT_50_)

## Discussion

### Stage dependent resistance

In all three species larvae showed the lowest tolerance to fresh and salt water and surprisingly nymphs and adults exhibited similar survival times, whereas in *C. bermudensis* nymphs are evidently the most tolerant stage. Schulte (1978) elicited differences between juvenile and adult ameronothrid mites with regard to seawater tolerance and Schuster (1979) argued that the found lower tolerance of immatures may be explained by their occurrence in upper zones of the littoral, above the habitats of adults. Adults and juveniles of the present fortuyniid and selenoribatid species occur in the same vertical zones of the intertidal and this may explain their equal tolerances. The low resistance of larvae may be caused by the interruption of larval/nymphal transition and the attendant inability to moult. None of the submersed individuals, neither larvae nor nymphs, did moult; therefore permanent inundation may have inhibited transition to the next stage and thus induced additional physical stress. However, nymphs survived longer than larvae although they were also unable to moult. But an increasing duration of juvenile stages with ontogeny has been observed in many Oribatida (Sǿvik and Leinaas 2003) and therefore stress caused by the inability to moult may impact later on nymphs, especially deuto- and tritonymphs.

### Resistance to fresh and salt water

Schuster (1966, 1979) observed that the type of water plays a major role in surviving inundation. He demonstrated that intertidal mites are more resistant to salt water while typical terrestrial oribatids are more tolerant to fresh water. This theoretic rule is true for *F. atlantica* and *A. inexpectatus* but not for *C. bermudensis*. The latter species is without a doubt a typical thalassobiont organism even though it is more tolerant to fresh water. This high freshwater tolerance is exceptional for an intertidal mite and may be a relic of an ancestral trait. Schuster (1966) hypothesized that terrestrial ancestors of intertidal mites first dwelled in freshwater habitats, then invaded brackish waters and finally colonized the marine littoral. A similar evolutionary history was postulated for the littoral Ameronothridae as well (Schulte and Weigmann 1977) and Schuster (1979) showed that *Ameronothrus maculatus* (Michael, 1882) also better tolerates fresh water, just like *Carinozetes*. But *A. maculatus* was classified as a transition species (Schulte and Weigmann 1977) and was recorded far inland along the shores of rivers (Schulte et al. 1975). *C. bermudensis* has never been detected away from the shore and/or at the edge of freshwater bodies, but it was found in the intertidal of marine ponds (personal observation), which show dramatically decreased salinity after long or heavy rainfall. This may be an indication that *Carinozetes* also made its evolutionary way to the intertidal environment via brackish waters and still is able to colonize habitats with at least periodically reduced salinities.

Furthermore, the differences in the tolerance to salt water shown by the three species, may reflect different levels of adaptation to the marine littoral environment. *F. atlantica*, displaying the highest resistance to salt water, possesses a very streamlined body shape and occurs predominantly in the lower zone of the eulittoral, *A. inexpectatus* is basically restricted to the median area of the intertidal, and *C. bermudensis* can mainly be found in the upper eulittoral zone. The lower a species occurs within the intertidal zone, the longer are the daily periods of tidal inundation, therefore a relatively elevated resistance to salt water, as shown in *F. atlantica*, may be advantageous for the colonization of the lower areas.

### Implications for hydrochorous dispersal

**Fig. 3:**
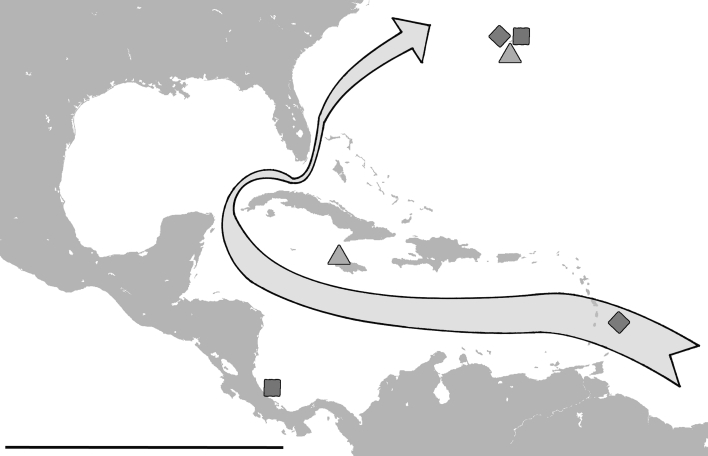
Geographic distribution of the genera *Alismobates*, *Fortuynia* and *Carinozetes* in the Caribbean and the Western Atlantic (findings on Barbados, Jamaica and Costa Rica are based on unpublished records). *Arrow* representing direction of Gulf Stream and thus possible route of hydrochorous dispersal. *Square*, *Alismobates*; *diamond*, *Fortuynia* and *triangle*, *Carinozetes*, *scale bar* = 2,000 km

Schatz (1991, 1998) already argued that long distance dispersal of flightless arthropods to oceanic islands can be assumed mainly hydrochorous. Peck (1994) provided first evidence by finding arthropods, oribatid mites included, in or near floating debris but some also directly in the sea. Coulson et al. (2002) demonstrated that even terrestrial arthropods with different biologies are able to survive in salt water for over 14 days and the authors further supposed that transoceanic dispersal by mites and Collembola is a common phenomenon. The intertidal oribatid mites of the present study are able to survive completely submerged in seawater for periods of a month and even longer. This ability and the fact that they are constantly exposed to the open ocean, qualifies these organisms as ideal candidates for hydrochorous dispersal. Moreover, the maximum survival times of particular specimens suggest that the lack of food rather than the lack of atmospheric oxygen was the main limiting factor. Considering the attachment to algae or other driftwood, providing food resources for the mites, survival times may exceed experimentally determined times by far. Nevertheless, in addition to the physical requirements, natural conditions must also be favourable to guarantee successful transport on the water’s surface. Darwin (1859) already mentioned that ocean currents can either act as isolating or as facilitating mechanisms for an island. The Gulf Stream is supposed to be an important factor for the colonization of the Bermuda islands by plants and animals (e.g. Thomas 2004; Schatz and Schuster 2012). This ocean current originates in the Caribbean and flows along the Central American and the southern North American shoreline with an average speed of 1.8 m per second before reaching the Bermudas. The intertidal oribatid mites of this archipelago are assumed to be derived from Caribbean and Central American shores (Pfingstl and Schuster 2012a, b), approximately 3,000 km away. This assumption is further supported by unpublished records of congeners in the Caribbean area (Fig. 3) and even if the species on Bermuda evolved in situ, their ancestors must somehow have reached the archipelago. Floating along the Gulf Stream from these supposed areas of origin to the coast of Bermuda would give a potential journey time of around 20 days. The median lethal times of *A. inexpectatus*, *F. atlantica* and *C. bermudensis* coincide with this travel time very well, whereas maximum survival times outrange this time by far. So theoretically half of the specimens would survive this journey. Of course the scenario mentioned above represents the ideal case and under natural conditions wind directions, vortices etc. may retard transport considerably. But even if travel time is doubled, maximum survival times of all three species still exceed this time. Therefore hydrochorous transport of *A. inexpectatus*, *F. atlantica* and *C. bermudensis* over long distances is clearly possible and should be assumed, for these mites, as the major mode of dispersal.

For terrestrial oribatid mites, bird-mediated transport is supposed to be another possible way of reaching and colonizing remote islands (e.g. Schatz 1991, 1998; Lebedeva and Lebedev 2008) but evidence is scarce and authors are of different minds about the importance of this mode of distribution. Lebedeva and Lebedev (2008) clearly favor the theory of birds being the basic suppliers of soil mites to isolated archipelagos but Coulson et al. (2009), performing a study on sea bird nests on Spitsbergen, could not support the assumption that these nests may facilitate microarthropod colonization via bird phoresy. Pugh (1997) also excludes bird-mediated transport of oribatid mites to oceanic islands, arguing that phoretic oribatid mites have not been recorded from either the Antarctic or the sub-Antarctic and that mites dispersed by birds usually show specific morphological adaptations to attach to the bird’s body. However, for a successful transport in the feathers of a bird, it is not just necessary to find its way on and off the plumage, it is also vital to survive the flight. The latter part may be the most problematic for intertidal mites and although Pugh (1995) demonstrated high desiccation resistances in the Antarctic intertidal *Halozetes marinus* and *H. littoralis*, the species from subtropic Bermuda are very vulnerable to desiccation (personal observation). Lebedeva and Lebedev (2008) reported that hydrobiont mites were absent from nests and plumage of the birds in their study and admitted that birds probably distribute to a greater degree oribatid mites more resistant to drying.

Another alternative to the transport along ocean currents would be aerial dispersal but experimental data on this issue are rare and considered controversial. Glick (1939) published a comprehensive study, in which insect traps were installed on airplanes in order to investigate aerial dispersal of arthropods. Acari were trapped on various occasions and in different altitudes, but nearly all specimens belonged to phoretic mite taxa, using insects as transport hosts. Gressitt and Yoshimoto (1974) used fine meshe aerial nets to investigate dispersal mechanisms in Alaska and they actually caught a few oribatid mite specimens drifting in the wind, whereas Coulson et al. (2003) performed a similar study but evidence of wind dispersal by mites was lacking. Another recent study, performed by Lehmitz et al. (2011), demonstrated that oribatid mites can be dispersed by wind up to at least 160 m above ground level but the majority of wind-dispersed mites consisted of species usually living in tree habitats. Pugh (2003) argued that powerful storms would be necessary to propel flightless arthropods long distances of a few 1,000 km but he also stated that these animals would not survive the attendant extreme conditions, such as low pressure and freezing temperatures at high altitudes. Nevertheless, every year the Caribbean and Bermuda are exposed to a series of tropical storms and hurricanes, therefore the possibility of aerial transport of intertidal mites should not be completely neglected.

However, the results of the present study clearly demonstrate that transport over considerable distances, on or in seawater, is feasible and that this mode of dispersal may be the most important for intertidal oribatid mites.

## References

[CR1] Coulson SJ, Hodkinson ID, Webb NR, Harrison JA (2002). Survival of terrestrial soil-dwelling arthropods on and in seawater: implications for trans-oceanic dispersal. Funct Ecol.

[CR2] Coulson SJ, Hodkinson ID, Webb NR (2003). Aerial dispersal of invertebrates over a high-Arctic glacier foreland: Midtre Lovénbreen, Svalbard. Polar Biol.

[CR3] Coulson SJ, Moe B, Monson F, Gabrielsen GW (2009). The invertebrate fauna of High Arctic seabird nests: the microarthropod community inhabiting nests on Spitsbergen, Svalbard. Polar Biol.

[CR4] Darwin CR (1859). On the origin of species by means of natural selection, or the preservation of favoured races in the struggle for life.

[CR5] Glick PA (1939). The distribution of insects, spiders and mites in the air. Techn Bull.

[CR6] Gressitt JL, Yoshimoto CM (1974). Insect dispersal studies in Northern Alaska. Pac Insects.

[CR7] Krisper G, Schuster R (2008). *Fortuynia atlantica* sp. nov., a thalassobiontic oribatid mite from the rocky coast of the Bermuda Islands (Acari: Oribatida: Fortuyniidae). Ann Zool.

[CR8] Lebedeva NV, Lebedev R (2008) Transport of oribatid mites to polar areas by birds. In: Bertrand M, Kreiter S, McCoy KD, Migeon A, Navajas M, Tixier M-S, Vial L (eds) Integrative acarology. Proceedings of the 6th European Congress. Montpellier, pp 359–367

[CR9] Lehmitz R, Russel D, Hohberg K, Christian A, Xylander WER (2011). Wind dispersal of oribatid mites as mode of migration. Pedobiologia.

[CR10] Peck SB (1994). Sea-surface (pleuston) transport of insects between islands in the Galápagos Archipelago, Ecuador. Entomol Soc Am.

[CR11] Pfingstl T, Schuster R (2012). First record of the littoral genus *Alismobates* (Acari: Oribatida) from the Atlantic ocean, with a redefinition of the family Fortuyniidae based on adult and juvenile morphology. Zootaxa.

[CR12] Pfingstl T, Schuster R (2012). *Carinozetes* nov. gen. (Acari: Oribatida) from Bermuda and remarks on the present status of the family Selenoribatidae. Acarologia.

[CR13] Pugh PJA (1995). Air-breathing littoral mites of sub-Antarctic South Georgia. J Zool Lond.

[CR14] Pugh PJA (1997). Acarine colonization of Antarctica and the islands of the Southern Ocean: the role of zoohoria. Polar Rec.

[CR15] Pugh PJA (2003). Have mites (Acarina: Arachnida) colonised Antarctica and the islands of the Southern Ocean via air currents?. Polar Rec.

[CR16] Pugh PJA, King PE, Fordy MR (1990). Respiration in *Fortuynia maculata* Luxton (Fortuyniidae: Cryptostigmata: Acarina) with particular reference to the role of van der Hammen’s organ. J Nat Hist.

[CR17] Schatz H (1991) Arrival and establishment of Acari on oceanic islands. In: Dusbábek F, Bukva V (eds) Modern acarology, vol 2. Academia, prague and SPB Academic Publishing, The Hague, pp 613–618

[CR18] Schatz H (1998). Oribatid mites of the Galápagos Islands—faunistics, ecology and speciation. Exp Appl Acarol.

[CR19] Schatz H, Schuster R (2012). First records of Lohmanniidae (Acari: Oribatida) from the Bermuda islands. Acarologia.

[CR20] Schulte G (1978). Die Küstenbindung terrestrischer Arthropoden und ihre Bedeutung für den Wandel des Ökosystems marines Felslitoral in unterschiedlichen geographische Breiten. Mitt Dtsch Ges allg angew Entomol.

[CR21] Schulte G, Weigmann G (1977). The evolution of the family Ameronothridae (Acari: Oribatei) II. Ecological aspects. Acarologia.

[CR22] Schulte G, Schuster R, Schubart H (1975). Zur Verbreitung und Ökologie der Ameronothriden (Acari, Oribatei) in terrestrischen, limnischen und marinen Lebensräumen. Veröff Inst Meeresforsch Bremerh.

[CR23] Schuster R (1966) Hornmilben (Oribatei) als Bewohner des marinen Litorals. Veröff. Inst. Meeresforsch. Bremerhaven, Sonderband II (6. Meeresbiol. Symposion, Bremerhaven 1965), pp 319–327

[CR24] Schuster R, Rodriguez JG (1979). Soil mites in the marine environment. Recent advances in acarology.

[CR25] Schuster R (1989). Transoceanic distribution of air-breathing littoral mites. Prog Acarol.

[CR26] Sǿvik G, Leinaas HP (2003). Long life cycle and high adult survival in an arctic population of the mite *Ameronothrus lineatus* (Acari, Oribatida) from Svalbard. Polar Biol.

[CR27] Thomas MLH (2004). The natural history of Bermuda.

